# Institutional Abuse, Neglect and Harm in UK Community Mental Health Services: A Scoping Review of the Peer‐Reviewed Evidence

**DOI:** 10.1111/hex.70403

**Published:** 2025-09-27

**Authors:** Bethan M. Edwards, Alan Meudell, Ellen Thomas, Eva Broeckelmann, Eva Roberts, Mark Farmer, Naomi Ghafoor, Sarah Markham, Catherine A. Robinson, Angela Sweeney, Sarah Carr, Michael Clark

**Affiliations:** ^1^ Service User Research Enterprise (SURE) The Institute of Psychiatry, Psychology and Neuroscience, King's College London UK; ^2^ Social Care and Society, Faculty of Biology, Medicine and Health The University of Manchester UK; ^3^ Independent Service User/Survivor Researcher UK; ^4^ Department of Biostatistics & Health Informatics The Institute of Psychiatry, Psychology and Neuroscience, King's College London UK; ^5^ Care Policy & Evaluation Centre, London School of Economics & Political Science London UK

**Keywords:** abuse, community, harm, mental health, neglect, patient safety, safeguarding

## Abstract

**Background:**

Statutory guidance relating to the prevention of institutional abuse, neglect and harm does not reflect the contemporary organisation of UK community mental health services. Historical associations with inpatient and residential settings have created a practice and conceptual gap despite lived experience testimony, inquiries and media reports attesting to the phenomena in community mental health services.

**Aim:**

To describe the peer‐reviewed evidence on the phenomena of institutional abuse and neglect associated with harm in UK community mental health services for adults of working age.

**Methods:**

We searched nine indexed databases for primary and secondary research (any methodology), theoretical papers and commentaries published between 2000 and 2025. We extracted and charted papers' substantive characteristics and findings, and conducted a descriptive synthesis to identify the phenomena's characteristics and potential causal factors.

**Findings:**

Twenty‐two papers met inclusion criteria, consisting of primary research utilising qualitative or observational methods (*n* = 12), secondary research (*n* = 6), lived experience narratives (*n* = 3) and a community consultation (*n* = 1). Institutional neglect was the principal phenomenon described, with gaps and inadequacies in accessing institutional provisions a primary mechanism. Associated harms included suicide, homicide and psychological harms. People diagnosed with a personality disorder, labelled ‘hard to engage’ and who self‐harm were amongst the population affected. Inter‐related potential causal factors spanned national, institutional and individual levels, with resources a primary factor.

**Conclusions:**

Our scoping review advances conceptual knowledge about the characteristics and potential causal factors of institutional abuse, neglect and harm in UK community mental health services. This provides a robust foundation for future research endeavours to inform safeguarding and patient safety policy and practice.

**Patient and Public Contribution:**

The review was conceptualised, led and conducted by lived experience researchers who are current and/or previous users of mental health services. A Lived Experience Advisory Group (LEAG) was involved in the review's synthesis, manuscript preparation and are review authors.

## 1 Introduction

### 1.1 Background

Institutional abuse and neglect were significant determinants of the deinstitutionalisation process that transformed UK mental health services from inpatient to community‐based [1, 2]. Today, NHS mental health bed numbers are at a record low, and the institutions and ‘asylums’ of the 19th and early 20th centuries have largely disappeared; however, abuse, neglect and harm continue to be associated with mental health services [3, 4, 5, 6].

In recent years, there has been a continuous stream of media exposés depicting severe institutional harms in English inpatient and residential mental healthcare settings, such as care homes [7, 8, 9]. Non‐residential community‐based mental health services have traditionally received media attention following incidents such as homicides. For example, the preventable homicides in Nottinghamshire NHS Foundation Trust [10, 11] highlight institutional failures constituting neglect, reminiscent of events leading to the death of Jonathan Zito 30 years ago [12].

Reflecting the emphasis in related fields (e.g., patient safety) on inpatient settings [13], abuse and neglect associated with community mental health services have historically not received such widespread media or academic attention, particularly where the person using or in need of services is the victim of harm. However, a plethora of first‐person accounts [6, 14, 15, 16], the extensive dissatisfaction and poor care reported by people using community mental health services [17, 18], the reports and briefings of third sector, non‐governmental organisations and the Parliamentary and Health Service Ombudsman [4, 19], as well as the UK Parliament's inquiry into community mental health services [20], suggest that these phenomena may have historically been under‐reported in this context. In the absence of prior evidence syntheses on the phenomena of abuse, neglect and harm perpetrated by community mental health services, this paper reports on a scoping review of the peer‐reviewed evidence.

### 1.2 Terminology

In this paper, we use the word institutional, akin to the then Department of Health's [21] ‘No Secrets’ guidance on the prevention of abuse. More recently, however, organisational is the preferred terminology used by the Care Act's (2014) statutory guidance [22]. We recognise that both words are often used interchangeably and acknowledge that institutional, in a mental health context, may be associated with hospitals, historically known as institutions. However, the terms institutional racism [23] and institutional sexism [24] are widely utilised and understood in the United Kingdom and are not associated with physical buildings or hospitals. Rather, institutional in these contexts refers to complex social structures at the meso level (e.g., the police, the NHS or the healthcare system) [25, 26]. It is in this manner that we use the word institutional, whilst recognising that there is a lack of consistency and consensus on terminology in this field.

### 1.3 Institutional Abuse, Neglect and Harm

Safeguarding adults who have care and support needs (including mental health needs) from abuse and neglect is enshrined in English law by the Care Act (2014). This is the main legislation for adult social care in England, with the United Kingdom's devolved administrations legislating in Scotland, Northern Ireland and Wales. The Care Act's (2014) legal framework is not confined to adult social care; its protections extend to adults who have care or support needs that are met (or should be met) by NHS, charity, community and independent (private) providers.

Institutional abuse is identified as a specific form of abuse by the Care Act's (2014) statutory guidance [27]. Like other forms of abuse (e.g., financial, sexual and physical), the Care Act (2014) itself does not provide a legal definition. Rather, statutory guidance briefly defines what it calls institutional abuse as ‘*neglect or poor professional practice*’ with institutional causal factors, including an institution's policies, procedures, structure and culture [27]. Only one example of abuse perpetrated by an institution (as opposed to an individual) is included in this guidance, and this pertains to a care home, that is, a community residential setting [27]. Statutory guidance, therefore, leaves mental health safeguarding practitioners with scant detail about how institutional abuse and neglect may manifest in non‐residential community mental health services.

Perhaps reflecting the historical association with inpatient settings, the lack of detail contained in, and the generic nature of, safeguarding statutory guidance (i.e., it is not mental health specific), there has been no prior review of the evidence on the topic of abuse, neglect or harm perpetrated or enabled by non‐residential community mental health services. Existing safeguarding evidence in this area is scarce, with evidence on *institutional* abuse and neglect deriving from research conducted in non‐mental health specific settings and populations [28, 29]. Further, evidence generated in relation to people with mental health needs has not specifically explored *institutional* abuse and neglect [30, 31, 32]. Nevertheless, this evidence indicates that knowledge about safeguarding policies, procedures and what constitutes abuse and neglect is variable, leading to inconsistent implementation and safeguarding practices [28, 29, 30, 31, 32]. For example, Johnson [29] identified that the majority of safeguarding professionals appeared not to consider cases when an institution (in particular the NHS or police), rather than an individual, was the perpetrator of abuse and neglect to be a safeguarding concern. Furthermore, Fennell [28] reported that NHS or health‐based staff make disproportionately fewer safeguarding referrals compared to staff working in other sectors, for example, social care or the police. Existing evidence also indicates that working‐age people using mental health services may not consider safeguarding as something that applies to themselves (e.g., assuming it is for children and older people) [30]. If they have had contact with safeguarding professionals and processes, their experiences are typically negative (e.g., due to lengthy processes and ‘passing the buck’ practices) [30].

### 1.4 Rationale and Aim

UK statutory guidance concerning the prevention of abuse and neglect perpetrated by institutions (as opposed to individuals) does not reflect the modern‐day organisation of mental health services, which are overwhelmingly non‐residential and community‐based. Safeguarding research is limited, with evidence indicating that abuse and neglect perpetrated by institutions (e.g., the NHS or Local Authority [LA]) as opposed to an individual, is less likely to be identified and addressed as a safeguarding issue. The lack of research and emphasis in statutory guidance on institutions as perpetrators or enablers is at odds with the abuse, neglect and harm reported by the media, patients, carers and non‐governmental organisations. In light of this, and to inform the direction of a future programme of research, we conducted a scoping review which aimed to:

- Identify and describe the peer‐reviewed evidence on the phenomena of institutional abuse and neglect associated with harm in UK community mental health services for adults of working age.

## 2 Materials and Methods

The Joanna Briggs Institute's (JBI) updated methodological guidance for scoping reviews was used alongside the PRISMA‐ScR extension to inform the conduct and reporting of this review [33, 34]. We developed an a priori protocol, which is available by contacting the corresponding author. Given their exploratory nature, scoping review protocols can be amended iteratively during the data extraction, analysis and presentation stages [35], and we highlight any deviations from the original protocol where relevant.

### 2.1 Research Questions

Three a priori research questions were developed, with a fourth developed post searching and eligibility checking, in response to the nature of papers identified:

1. *What are the characteristics and findings of papers published on the phenomena of institutional abuse and neglect associated with harm in UK community mental health services for adults of working age?*
2. *How has institutional abuse and neglect been defined or conceptualised by these papers?*
3. *Post hoc: What characteristics and potential causal factors of institutional abuse and neglect are reported by these papers?*
  ○ *When does it happen? How does it happen? Who does it affect? What harms are associated with it? Why does it happen?*
4. *What evidence gaps, if any, exist?*

### 2.2 Search Strategy

Our search strategy was developed with the support of two subject librarians, with the following indexed databases searched on the 27th and 28th of September 2023: EMBASE, Medline, PsycINFO, CINAHL, HMIC, AMED, Social Policy and Practice, ASSIA and Social Services Abstracts. We updated our search using the same strategy in five databases on the 17th of February 2025, with searches conducted in ASSIA and Social Services Abstracts completed on the 4th of March 2025. Our search string consisted of keywords and MeSH headings, amended according to each individual database's search functions. Supporting Material 2 consists of the Boolean search strategy used for Medline. We additionally identified potentially relevant papers during full‐text screening and through expert recommendation, which were obtained for full‐text screening.

### 2.3 Eligibility Criteria

Papers were included if they reported on: (1) Institutional abuse and/or neglect associated with harm (Table 1); in the context of (2) Non‐residential community or outpatient‐based mental health services in the United Kingdom; in relation to the population of (3) Adults of working age (18–66 inclusive). Additional criteria consisted of: (4) An empirical or non‐empirical paper published in a peer‐reviewed journal, for example, primary research (any methodology), secondary research (any methodology), commentaries, editorials and theoretical and conceptual papers; (5) Written in English; (6) Dated from the year 2000 onwards, coinciding with then Department of Health's guidance on protecting vulnerable adults from abuse, ‘No Secrets’ [21]. Further eligibility criteria can be found in Supporting Material S1.

**Table 1:** Definition of phenomenon (concept) utilised.

Include	Papers that report on the phenomena of institutional abuse and/or neglect associated with harm:
	*Institutional* was defined using the micro‐meso‐macro framework at the meso level [25, 26]. Mental health and social care commissioners and providers, including Integrated Care Boards (ICBs), Clinical Commissioning Groups (CCGs), Local Authorities (LAs), NHS Trusts and Health Boards was in scope, including the services which they commission or provide. *Institutional abuse* was defined as acts of abuse that may be sexual, financial, emotional, psychological, physical and discriminatory, as per the Care Act (2014), with evidence of potential institutional causal factors. *Institutional neglect* was defined as a failure to meet health and care needs in a timely manner, with evidence of potential institutional causal factors. *Institutional causal factors* were defined as causal factors at the institutional/meso level (e.g., leadership, an organisation's resources, staffing, culture, policies, processes and structure). *Harm* was defined broadly, inclusive of physical and psychological harms of differing severit (e.g., suicide, homicide, deterioration in physical and mental health and readmission).
Exclude	Papers that report on:
	Abuse and/or neglect that is not associated with harm as defined above. Abuse and/or neglect in the absence of potential institutional causal factors (e.g., the perpetrator is an individual, or no institutional causal factors are described).

### 2.4 Evidence Screening and Selection

EPPI‐Reviewer 4 [36] was utilised to support the screening and evidence selection process, which occurred in two stages. Stage 1 consisted of two reviewers (B.E. and A.M.) screening all titles and abstracts to determine if the evidence was relevant, potentially relevant or not relevant. After the first double screening of 100 titles and abstracts, both reviewers met to evaluate the suitability of the developed eligibility criteria and compare findings. This led to two refinements to our review protocol to enhance our understanding of the phenomena and context. Firstly, potential causal factors at the institutional (meso) level (e.g., an organisation's leadership, resources, staffing and culture), individual (micro) level (e.g., knowledge, attitudes and skills) and national (macro) level (e.g., national policy and resources) were proposed, with papers having to report on institutional causal factors to be included. Secondly, we defined abuse and neglect in relation to harmful outcomes, for example, suicide, homicide, deterioration in physical and/or mental health and admission to hospital. This helped distinguish between the everyday use of the words ‘abuse’ and ‘neglect’, not in the context of the Care Act (2014). It additionally reflects the use of the word harm (as perhaps a more concrete concept rather than abuse and neglect) by Scottish policy and legislation (e.g., the Adult Support and Protection (Scotland) Act 2007) [37]. Reviewers met regularly during the first stage of screening and selection to discuss disagreements, which were resolved through discussion and consensus. Stage 2 consisted of obtaining in full, evidence deemed relevant or potentially relevant, with full texts read by both reviewers (B.E. and A.M.), with include and exclude as potential outcomes. Disagreements were resolved through discussion and consensus.

### 2.5 Data Extraction and Charting

Data were extracted on the included evidence's substantive characteristics (e.g., aim and methods) as well as data of relevance to the review questions (e.g., definitions or conceptualisations of the phenomena). We additionally extracted the findings sections of included evidence to conduct a descriptive synthesis (detailed below). Extraction was initially performed by B.E., who conducted a second cross‐checking of all extracted data to verify its accuracy before submission.

### 2.6 Descriptive Synthesis

We conducted a descriptive thematic synthesis of included papers' findings sections, to answer our third research question, to advance conceptual knowledge about the phenomena. Where clearly identified as such, findings relating to non‐community and residential settings were excluded from our synthesis, as was data that was not relevant to our review questions. The synthesis occurred in six stages (Table 2).

**Table 2:** Descriptive thematic synthesis process.

Stage	What?	Whom?
1. Familiarisation	Initial reading of findings sections. Making memos about initial impressions and reflections.	B.E.—all included papers.
		E.B., E.T., N.G., S.M. and M.F. and one additional LEAG member—3 papers each.
2. Initial coding of data segments	Inductive coding of data segments/chunks in Microsoft Word or on hard copy.	B.E.—all included papers.
		E.B., E.T., N.G., S.M. and M.F. and one additional LEAG member—3 papers each.
3. Initial development of descriptive categories	Online workshop to (1) present and discuss Stages 1 and 2 and (2) develop initial descriptive categories using a Zoom whiteboard by grouping codes based on similarities.	B.E., E.B., E.T., N.G., S.M. and M.F. and one additional LEAG member.
4. Initial development of coding framework	Reviewing and refining descriptive codes and categories into a coherent NVivo coding framework.	B.E. and A.M.
5. Coding the entire dataset and refining the framework	Coding and categorising in NVivo using an initial coding framework. Iterative refinements made to the framework as required.	B.E.
6. Reporting the analysis	Write up in the form of a peer‐reviewed publication.	All authors.

### 2.7 Patient and Public Involvement

This was a lived experience‐led (survivor) study [38] and as such, the review was conceptualised, led and conducted by lived experience researchers (B.E., A.M., E.T., E.B., E.R., N.G., S.M., and M.F.) who are current and/or previous users of mental health services. Our Lived Experience Advisory Group (LEAG), consisting of 6 individuals with lived experience of the review topic, met on four occasions, with additional one‐to‐one meetings arranged as needed. The focus of LEAG meetings was on the review's descriptive synthesis. All LEAG members elected to participate in the synthesis during Stages 1–3, with bespoke group and individual training provided by the lead author, B.E., to enable participation in the analysis. Members were again offered the opportunity to contribute to the writing of this manuscript as members of the analysis team, and those who took up this opportunity are review authors.

## 3 Findings

**Figure 1:**
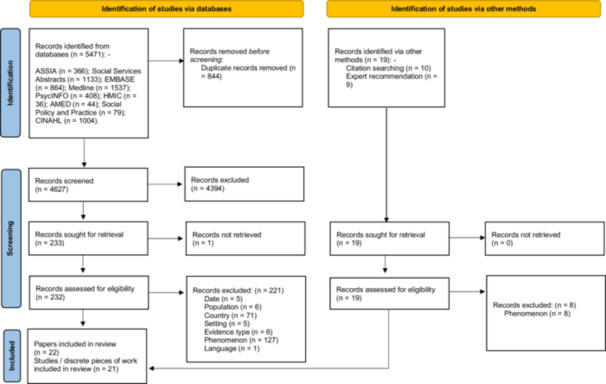
PRISMA 2020 Flow Diagram.

Twenty‐two papers met inclusion criteria, reporting on 21 studies or discrete pieces of work (Figure 1). Significantly, no papers specifically aimed to investigate the phenomena of institutional abuse and neglect associated with harm, despite our search strategies that took an inclusive approach to variations in terminology (e.g., organisational, systemic or institutional; harm, abuse or neglect). Included papers met inclusion criteria by reporting on our definition of neglect and/or abuse with institutional causal factors and associated harmful outcomes (Supporting Material 1).

### 3.1 Evidence Characteristics and Findings

Table 3 presents the substantial characteristics and findings of included papers. These derive from multiple broadly defined fields or disciplines, with some overlap, consisting of:

1. Patient safety with a NHS or health services focus (*n* = 8) [13, 39, 40, 41, 42, 43, 44, 45];
2. Safeguarding, with a social care or social work focus (*n* = 3) [30, 46, 47];
3. Coronial law, involving the analysis of Prevention of Future Death (PFD) reports (*n* = 2) [48, 49];
4. Lived experience, including research and narrative accounts by individuals who identify as mental health service users, carers or family members (*n* = 3) [3, 30, 47], and professionals (*n* = 1) [50]; and
5. Health services and delivery research, with primarily an NHS focus on the topic of suicide and self‐harm (*n* = 7) [51, 52, 53, 54, 55, 56, 57].

Papers were published between the years 2006 and 2025, with the majority (*n* = 20) from 2018 onwards. Geographically, lead authors were typically located in Northern (e.g., Manchester, Liverpool and Leeds) and Southern (e.g., London and Essex) England. Where applicable, participants (or data relating to individuals, e.g., from inquiry reports) were residing primarily in England; however, a number of papers did not report on location beyond ‘the UK’ [39, 40, 41, 42, 43].

Twelve papers reported on the results of qualitative or observational primary research, with semi‐structured interviews the principal method of data generation (*n* = 8) [30, 39, 42, 43, 44, 53, 54]. Participants (or data relating to individuals) comprised people who used, were attempting to use, or had used mental health services (*n* = 9) [30, 40, 41, 43, 44, 51, 52, 54]; informal caregivers (*n* = 7) [30, 40, 41, 43, 44, 52, 54] and professionals (*n* = 8) [30, 39, 40, 41, 42, 44, 53, 54]. Six papers reported on secondary research, with documentary analysis the primary method (*n* = 5). Documents analysed comprised Safeguarding Adults Reviews (SAR) [46], PFD reports [48, 49], Patient Safety Incidents (PSIs) [45] and Mental Health Homicide Inquiries (MHHIs) [57]. An additional four papers did not report on primary or secondary research. Three consisted of lived experience narratives or reports from a personal (*n* = 2) [3, 47] and professional (*n* = 1) [50] perspective. One paper reported on a community consultation exercise [56].

Only four papers had a specific focus on the context of community mental health services [13, 44, 45, 55], and one in the context of an outpatient service (A&E) [54]. Papers' context typically consisted of both community and inpatient mental health services, with four papers pertaining to a broader range of services, including safeguarding teams, housing and the police [30, 46, 48, 49].

### 3.2 Existing Definitions and Conceptualisations

No definitions or conceptualisations of institutional abuse and neglect were reported by included papers; however, alternative concepts and theoretical frameworks were identified (Table 3). The most frequently utilised was the Yorkshire Contributory Factors Framework (YCFF) or YCFF—Mental Health (YCFF‐MH) to inform data generation and/or its analysis in five patient safety papers [13, 40, 42, 43, 45]. The YCFF‐MH identifies 20 factors contributing to PSIs in both community and inpatient mental health services [43].

**Table 3:** Evidence characteristics and key findings.

Short reference and journal	Discipline/Field	Key concepts/theories	Aim	Design and Method(s)	PPI	Participants/Population	Setting and country/region	Relevant key findings (Summary)
**Albutt et al. (2021)** International Journal of Mental Health Nursing	Patient safety	YCFF‐MH.	To explore mental health professionals' views on patient safety issues in community and inpatient mental health services.	Qualitative Semi‐structured interviews Framework analysis guided by the YCFF‐MH	Not reported.	*n* = 14 mental health professionals.	Mixed—Community and inpatient mental health services. Participants: the United Kingdom. Research team: Leeds and Bradford, England.	The most common safety issues identified include: (1) Safety culture (e.g., feeling unable to raise concerns); (2) Communication systems; (3) Service user factors (e.g., increased acuity); (4) Service processes (e.g., waiting lists); and (5) Unmanageable workloads PPI.
**Averill et al. (2023)** Health Expectations	Patient safety	YCFF‐MH used to illustrate case study.	To review patient safety literature on community‐based mental health services and identify conceptual and empirical challenges associated with understanding safety in this setting.	Narrative review Informed by 71 sources, including peer‐reviewed and grey literature.	No PPI.	N/A	Community‐based mental health services. Research team: London and Oxford, England.	Conceptual challenges discussed include: (1) A lack of patient safety research pertaining to community mental health services, but evidence is emerging; (2) Service user perspectives have not traditionally been prioritised; (4) Challenges measuring harm which are cumulative, less visible and not immediate; (6) Challenges weighing up one potentially harmful event (e.g., MHA detention) with another (e.g., delaying/not conducting MHA).
**Averill et al. (2024)** BMC Health Services Research	Patient safety	Patient safety in the context of community‐based mental healthcare.	To explore conceptualisations of ‘patient safety’ in community‐based mental health services.	Qualitative Semi‐structured interviews and focus groups. Thematic analysis	Seven individuals provided PPI input for the study questions, topic guide, piloting and research information sheets.	*n* = 43 (service users *n* = 13; carers *n* = 12; professionals; *n* = 18)	Community‐based mental health services, including GP. Participants: 1 NHS England trust and online recruitment. Research team: London, England.	Four primary themeswere developed: (1) ‘*Systemic inertia: threats to safety’* (p4); (2) ‘*Managing the risks service users present’* (p8); (3) ‘*More than responding to risks: everyone plays a role in creating safety*’ (p9); and (4) ‘*The goals of “safety”: our destination is not yet in sight’* (p10).
**Averill et al. (2025)** Psychological Medicine	Patient safety	YCFF‐MH.	To characterise the nature of PSIs, contributory factors and solutions in community‐based mental health services.	Documentary analysis and descriptive statistics. Content analysis and descriptive statistics of PSI reports extracted from the England and Wales National Reporting and Learning System (NRLS) from the year 2019.	Not reported.	N/A. 1443 PSI met inclusion criteria.	Community‐based mental health services, consisting of primary and secondary care. 22 English NHS trusts.	PSIs were categorised in relation to: investigations, documentation, referral, communication, administration, treatment and procedure, medication, diagnosis and assessment, and ‘service influence unclear’. The majority of PSI's were categorised as ‘service influence unclear’. Contributory factors included unmanageable workloads, management of staff, staffing levels, and communication systems.
**Aves (2024)** International Journal of Mental Health Nursing	Survivor/Lived experience	Iatrogenic harm	To provide a first‐person perspective of experiences of harm associated with mental health services.	Lived experience narrative	Not reported.	N/A. First‐person account of someone who has used mental health services.	Does not specify community or inpatient. Relates to the United Kingdom; no further details provided.	Describes multiple harmful experiences, including: (1) ‘*I was stripped of all authority over my life and internal circumstances’* (p669); (2) ‘*To have any chance of receiving support, I had to play a complex set of games’* (p669); (3) ‘*Replication of childhood trauma’* (p670); (4) ‘*They broke the rules, but I got the blame’* (p670); and (5) ‘*Long‐term harms of mental health services…. The reality of service avoidance’* (p671).
**Baker, D., Fidalgo et al. (2023)** International Journal for Crime, Justice and Social Democracy	Coronial law	N/A	To examine mental health‐related deaths in community, outpatient, inpatient and custodial settings over a 10‐year period.	Documentary analysis using a thematic approach of 221 PFD reports issued for deaths that occurred between October 2010 and March 2020	Not reported.	N/A. Deaths that resulted in a PFD report, which the Judiciary Service classified as a mental health‐related death.	Mixed—Deaths of individuals who were in contact with community‐based and inpatient mental health services, as well as indivduals who had never been in contact with formal mental health services. Deaths in England and Wales. Research team: Liverpool, England.	Suicide was recorded in 51% of deaths, a narrative conclusion in 17% and ‘suicide with factors’ in 18%. Themes identified relate to organisational and structural factors that contributed to deaths, including deficiencies in the provision of care (72% of PFD reports), communication (55%) and policy (26%).
**Baker, D., Lucy, et al. (2023)** Health, Risk and Society	Coronial law	Organisational learning; organisational irresponsibility	To examine organisational responses to PFD reports and whether lessons are learnt.	Documentary analysis of 210 institutional responses to 214 PFD reports associated with mental health deaths between 2010 and 2020. Analysis was inductive and thematic.	Not reported.	N/A	Mixed—Deaths of individuals who were in contact with community‐based and inpatient mental health services, as well as individuals who had never been in contact with formal mental health services. Organisations in England and Wales. Research team: Liverpool, England.	NHS Trusts (39%), Government agencies (18%) and Clinical Commissioning Groups (CCGs) (7%) were most frequently the recipients of PFD reports, with Local Authorities (LAs) receiving 5% and the CQC 3%. LAs responded to 66%, with NHS Trusts responding to 47%. Three themes were identified: (1) Generic responses (with organisations not addressing issues contained in PFDs; (2) Citing exiting policy, which would not have prevented the deaths; and (3) Shifting the blame to other organisations.
**Baker, J. et al. (2019)** International Journal of Nursing Studies	Patient safety; quality	Unsafestaffing	To investigate how staffing and skill mix affect mental healthcare safety and quality.	Qualitative Semi‐structured interviews Thematic analysis	Not reported.	*n* = 21 mental health professionals.	Mixed—Community‐based and inpatient mental health services. Participants: UK. Research team: Leeds, England.	Recurring, over‐arching theme: ‘*The “vicious cycle” of unsafestaffing’* (p3). Major components of this cycle are described under the themes: (1) Understaffing (lack of resources to provide safe care); (2) Chronic understaffing (the consequences of understaffing, which make it worse); and (3) Unsafestaffing (staffing that compromises safe care).
**Berzins, Baker, J. et al. (2018)** Health Expectations **Berzins, Louch, et al. (2018)** BMC Health Services Research	Patient safety	**Berzins, Baker, J. et al. (2018)**: YCFF used as a theoretical framework to inform data analysis.	**Berzins, Baker, J. et al. (2018)**: To identify safety issues in UK mental health care from the perspective of service users, carers and professionals. **Berzins, Louch, et al. (2018)**: To explore experiences of raising safety concerns from the perspective of mental health service users and carers.	Mixed‐methods Cross‐sectional author‐designed survey, generating quantitative and qualitative data. Descriptive statistics, with qualitative data coded inductively and mapped onto the YCFF.	Co‐author with lived experience of mental distress/illness. Input from others with lived experience into the design and content of the survey (not reported who or how many involved).	*n* = 185 (*n* = 90 health professionals; *n* = 77 service users; *n* = 18 carers)	Mixed—Community‐based and inpatient mental health services. Participants: UK. Research team: Leeds, England.	**Berzins, Baker, J. et al. (2018)**: Safety issues identified include: (1) Individual staff factors (e.g., poor attitudes, burn‐out); (2) Service process factors (e.g., high support thresholds, premature discharge from hospital); and (3) Management of staff and staffing levels (e.g., low staffing levels, high use of agency/bank). **Berzins, Louch et al. (2018)**: 77% of service users and carers reported it was difficult or very difficult to raise safety concerns. Reasons for difficulties included: (1) Services not listening; (2) Concerns about repercussions; (3) The process of raising concerns; and (4) difficulties raising concerns whilst being mentally ill (14%).
**Berzins et al. (2020)** Health Expectations	Patient safety	YCFF‐MH used as a theoretical framework to inform data analysis.	To explore service users and carers' perceptions of what constitutes a safety issue.	Qualitative Interviews Framework analysis using the YCFF‐MH—deductive and inductive approach.	Discussions on social media informed the aims of the study.	*n* = 13 service users; *n* = 7 carers	Mixed—Community‐based and inpatient mental health services. Participants: UK. Research team: Leeds and Bradford, England.	Most frequently reported safety issues include: (1) Safety culture (e.g., physical safety prioritised over psychological safety); (2) Staff factors (e.g., skills and attitudes towards personality disorder diagnosis); (3) Patient factors (e.g., prior experiences of trauma); (4) Staff management and levels (e.g., time‐limited interventions due to understaffing); and (5) Service processes (e.g., difficulties accessing support).
**Carr et al. (2019)** Health & Social Care in the Community	Social care/safeguarding Survivor/lived experience	Safeguarding, Care Act (2014)—Disability hate crime.	To explore experiences and understanding of targeted violence and abuse (disability hate crime).	Qualitative, multiple methods. Survivor/lived experience‐led research. Interviews (service users); focus groups (mental health and safeguarding practitioners); 2 x Twitter discussions.	Led and conducted by lived experience researchers. Input from an advisory group when developing interview questions (no details about membership).	*n* = 21 service users; *n* = 2 caregivers; *n* = 46 practitioners working in statutory services (e.g., police, mental health services, safeguarding etc); *n* = 724 Twitter responses.	Mixed—Community‐based and inpatient mental health services and additional stautory services. Participants: England. Research team: London, England.	Social deprivation, a reduction in statutory services and poor housing were considered to increase the likelihood that mental health service users experience disability hate crime. Service users identified the inadequacy, absence and/or fragmented nature of mental health and safeguarding services' responses to disability hate crime. Practitioners identified the impact that ‘*buck passing*’, ‘*blame cultures*’ and ‘*fear of speaking up*’ (p e786) had on safeguarding responses.
**Deshpande et al. (2025)** British Journal of Psychiatry	Health services and delivery (mental health homicides)	N/A	To examine the characteristics of Mental Health Homicide Inquiry (MHHI) reports.	Documentary analysis using the Ready materials, Extract data, Analyse data, Distil (READ) approach.	Not reported.	162 MHHI reports published on the NHS England website between 2013 and 2023.	Does not specify community or inpatient. Reports relating to MHHI in England. Research team: Southampton, England.	52% of perpetrators had a Schizophrenia diagnosis, 82% were male and 52% were ‘*non‐adherent*’ (p2) with service contact and treatment. The majority (86%) of victims were known to the perpetrator. Root causes and contributory factors included: (1) Patient factors and (2) Care and service delivery problems (e.g., deficits in assessment, treatment (including managing risk), care planning and the CPA, communication, documentation and management).
**Foss (2023)** The Journal of Adult Protection	Social care/safeguarding	Safeguarding, Care Act (2014); Mental Health Act 1983.	To examine the use of the Mental Health Act (MHA) 1983 in relation to safeguarding adults at risk of abuse and neglect.	Documentary thematic review of *n* = 69 SARs and 1 Adult Practice Review (APR) published between 1 April 2015 and 1 June 2021.	Not reported.	SARs and APRs pertaining to adults and older adults subject to the MHA.	Mixed—Community‐based and inpatient mental health services and additional statutory services. SARs and APRs in England and Wales. Research team: Keele, England.	60% of SAR/APRs were classified as ‘self‐neglect’, 14% as neglect and 7% as organisational neglect. 37% reported a reduction in service provision or delay. 57% related to someone labelled ‘hard to engage’. Failures were identified including not taking the person's views into consideration, poor assessment practices, and not facilitating engagement with the person subject to the SAR/APR.
**Laskaris (2023)** The Journal of Adult Protection	Social care/safeguarding Survivor/lived experience	Safeguarding, Care Act (2014); Mental Capacity	To explore the impact of the misappropriation of the presumption of mental capacity.	Lived experience narrative from the perspective of a parent.	Led and written by someone with lived experience. No further PPI reported.	N/A an account of a parent based on their son, Christopher's experiences of mental health and social care services.	Mixed—Community‐based and inpatient mental health services. Paper relates to England.	Multiple significant failures by services and professionals described, which led to Christopher's murder. Outlines how the law was consistently misunderstood and misapplied, including the statutory presumption of capacity. Describes how Christopher was considered to be ‘self‐neglecting’ due to a lack of appropriate support.
**Kapur et al. (2016)** Lancet Psychiatry	Health services and delivery (suicide and self‐harm)	N/A	To explore the association between suicide and the implementation of service changes and organisational factors.	Cohort study using data from the National Confidential Inquiry into Suicide and Homicide by People with Mental Illness (NCISH). Statistical analysis	Not reported.	*n* = 19,248 who died by suicide aged 10 and over who had contact with mental health services within 12 months of their deaths between 1997 and 2012.	Mixed—Community‐based and inpatient mental health services. People who died by suicide in England. Research team: Manchester, England.	A lower suicide rate (incidence rate ratios ranged from 0.71 to 0.79, *p* < 0.0001) was associated with the implementation of service changes consisting of staff training, improved community services and the implementation of national policy and guidance. Non‐medical staff turnover (Spearman's *r* = 0.34, *p* = 0.01) correlated with suicide rates.
**O'Keefe et al. (2021)** BJPsych Open	Health services and delivery (suicide and self‐harm)	N/A	To explore the experiences of patients, carers and professionals of treatment for self‐harm in A&E departments.	Qualitative Focus groups and semi‐structured interviews. Framework analysis, with a framework developed inductively.	A Lived Experience Advisory Panel (LEAP) contributed to the development of interview questions. One member is a co‐author and was involved in the analysis of data.	*n* = 19 service users, *n* = 8 carers, *n* = 15 generalist emergency department practitioners and *n* = 37 liaison psychiatry practitioners.	A&E—Outpatient‐based service. Service users and carers: England. Practitioners: London and South West England. Research team: London, England.	An overarching theme was identified: ‘*The wider system is failing people who self‐harm: they often only access crisis support as they are frequently excluded from services, leading to unhelpful cycles of attending the emergency department’* (p2). Additional themes describe: (1) Feelings of powerlessness and hardened attitudes amongst practitioners; (2)Patients feeling judged, exacerbating feelings of distress; and (3) Fear of blame was common amongst practitioners.
**Punton et al. (2022)** PLOS One	Health services and delivery	N/A	To explore the experiences of young adults of waiting lists for mental health services.	Qualitative Semi‐structured interviews. Interpretive phenomenological analysis.	Not reported. All participants provided feedback on developed themes.	*n =* 7 young adults who have experience of being on a waiting list for mental health support (19–22).	Community‐based mental health services. Participants: England (North East and Midlands) and Scotland. Research team: Newcastle, England.	Themes identified the following impacts: (1) Deterioration in mental health and ability to function day to day (e.g., self‐care, work and education) (2)The development of negative beliefs, thoughts and emotions; and (3) Having to rely on others and alternative sources of support.
**Seager (2006)** Psycholanalytic Psychotherapy	Psychotherapy/Psychology	Psychological safety	To propose and develop a psychoanalytically informed concept of ‘psychological safety’.	Professional narrative	Not reported.	N/A	Mixed community‐based and inpatient mental health services. Author: Essex, England.	Argues that a culture of ‘*mindless iatrogenic breaches and ruptures of attachments and containment*’ (p269) is present in mental health services enacted by referral/admission criteria, discharge policies, staff rotations, appointment systems, treatment quotas, bureaucratic systems, service cutbacks and reconfigurations. Maintains that these create an environment that is psychologically unsafe, insecure and toxic.
**Quinlivan, Gorman, Marks (2023)** BJPsych Open	Health services and delivery (suicide and self‐harm)	N/A	To examine barriers and facilitators to psychological therapy and aftercare following self‐harm amongst people who present to hospital.	Qualitative, pragmatism. Interviews Thematic analysis	Two members of the study advisory group are co‐authors and were involved in the interpretation of data.	*n* = 51 Liaison psychiatry practitioners (e.g., mental health nurses and consultant liaison psychiatrists).	Mixed community‐based and inpatient mental health services. 32 randomly selected hospitals in England. Researchers: Manchester, England.	Barriers led to a ‘*cycle of despair*’ (p3), waiting lists and increased likelihood of patient harm. Barriers included: (1) Difficulties accessing services do to exclusionary thresholds; (2)Perceptions of psychiatric versus social crises; (3) Insufficient bed numbers; (4) Silo working and poor relationships; (5) Bureaucratic processes; and (6) Skill erosion.
**Quinlivan, Gorman, Monaghan (2023)** BJPsych Open	Health services and delivery (suicide and self‐harm)	N/A	To explore experiences of accessing psychological therapies after self‐harm.	Qualitative, critical realism. Survey: open, closed and free text options. Thematic analysis	Patient and carer advisory group involved in all aspects of the research process and are co‐authors.	*n* = 128 service users, 23 carers.	Mixed community‐based and inpatient mental health services. Participants: Recruited through 16 mental health trusts in England and social media. Researchers: Manchester, England.	Themes identified relate to experiences of accessing psychological therapy after self‐harm and suggestions for improving care. The former consisted of: (1) ‘*Too little, too late*’ (p3); (2) ‘*Feeling like a non‐person’* (p4); (3) ‘*Challenging access to psychological therapies*’ (p4); and (4) ‘*Exclusion, rejection and punitive treatment*’ (p5).
**Tannerah et al. (2024)** Health Expectations	Health services and delivery	N/A	To identify inequalities in mental health care and support experienced by Muslims from ethnically and racially minoritised communities.	Consultation Notes and illustrations taken during consultation exercises. Reflexive thematic analysis.	Not reported. Paper reports on the outcomes of a PPI consultation.	*n* = 35 Muslim and ethnically minoritised consultees.	Not specified. Participants and research team: Liverpool, England.	Themes identified included: (1) Overmedicalised models of care, which were not compatible with consultees' culture or beliefs, including holistic care; and (2) A ‘*broken cycle of trust’* (p4) characterised by stigma, defensive practices and a lack of safety.

Abbreviations: APR, Adult Practice Review; CCGs, Clinical Commissioning Groups; CPA, Care Programme Approach; CQC, Care Quality Commission; LA, Local Authority; MHA, Mental Health Act; MHHI, Mental Health Homicide Inquiry; N/A, Not applicable; PFDs, Prevention of Future Deaths; PPI, Patient and Public Involvement; PSI, Patient Safety Incident; SARs, Safeguarding Adults Reviews; YCFF, Yorkshire Contributory Factors Framework; YCFF‐MH, Yorkshire Contributory Factors Framework—Mental Health.

### 3.3 Descriptive Synthesis: Characteristics and Potential Causal Factors

Papers primarily described or reported on our definition of institutional *neglect*, with specific examples of institutional *abuse* identified in the context of institutional *neglect*. Our descriptive synthesis, therefore, scoped the characteristics and potential causal factors of institutional neglect and, by extension, instances of institutional abuse.

#### 3.3.1 When Does Institutional Abuse and Neglect Happen?

We identified five broadly defined intersecting institutional provisions and/or processes that were recurrently associated with the phenomena's occurrence (see Table 4 for examples and illustrative quotes). These consisted of:

1. Assessment and diagnosis (including tests and investigations) [3, 13, 43, 45, 46, 47, 56, 57];
2. Care and/or treatment (including its planning and co‐ordination) [13, 39, 42, 44, 45, 46, 52, 53, 54, 56, 57];
3. Access or admission (to provision) [3, 13, 30, 39, 40, 42, 43, 44, 45, 46, 48, 52, 53, 54, 55, 56];
4. Discharge and transitions of care [40, 42, 44, 45, 46, 47, 52, 57] and
5. Communication and documentation [3, 30, 40, 44, 45, 46, 48, 49, 52, 54, 56, 57].

Access or admission to assessment, care and/or treatment was most frequently associated with the phenomena in the identified evidence. Difficulties pertained to accessing a range of services, including community mental health teams (CMHTs) [3, 13, 39, 42, 43, 44, 46, 52, 53, 54], crisis services [40, 42, 43, 44, 46, 53], NHS talking and psychological therapies [13, 48, 52, 53], A&E and liaison psychiatry [43, 54], in addition to inpatient services [46, 53] and Mental Health Act (MHA) assessments [45, 46], with implications for meeting a person's needs safely in the community.

The chronicity of neglectful and abusive provisions/processes was particularly evident in papers written from a lived experience perspective [3, 47], that analysed SARs [46] and PFD reports [48], as well as papers reporting on the experiences of people who are racially or culturally minoritised [56]. Rather than being an isolated incident leading to harm, this evidence demonstrated that an individual or a minoritised community, with mental health needs, may experience a series of institutional provisions/processes that are neglectful and/or abusive over the duration of months and years. A minority of patient safety papers highlighted the cumulative, longitudinal latent nature of safety problems leading to harm in community mental health services, which were contrasted with more immediate or observable safety incidents in a hospital setting [13].

#### 3.3.2 How Does Institutional Abuse and Neglect Happen?

Papers included in this review indicate that how the phenomena happen is complex and multifactorial. For example, it can manifest through multiple neglectful and abusive provisions and/or processes across several institutions or services within a person's system of care (e.g., inpatient providers, community secondary care services, approved mental health professional [AMHP] providers and the police), over a sustained duration of time [6, 30, 46, 47, 48, 49].

In the context of this complexity, we identified three primary ways (mechanisms) in which the phenomena occur (see Table 4 for examples and illustrative quotes). Firstly, we identified that institutional *neglect* occurred through *gaps, absences or omissions*, whereby an institutional provision/process was not provided, actioned or performed, or significant elements were missing. Secondly, it consisted of institutional provisions/processes that were *inadequate or inappropriate*, that is, something was provided but it was of poor quality or was provided in an untimely way, with the person's needs remaining unmet. A third mode was characterised by *dehumanising and discriminatory* institutional provisions/processes. In these instances, provisions not only failed to meet a person's needs through gaps, omissions or inadequacies (institutional *neglect*), but additionally appeared to meet our definition of institutional *abuse*, namely emotional, psychological, physical and/or discriminatory (Supporting Material 1).

**Table 4:** Institutional provisions/processes associatated with the phenomena's occurrence and identified examples (when and how does institutional abuse and neglect happen?).

Institutional provision examples and illustrative quotes	Contributing papers
**1. Assessment and diagnosis (including tests and investigations):**	[3, 13, 43, 45, 46, 47, 56, 57]
Gaps, absences or omissions in provision: Assessments (e.g., risk, Section 117, MHA and MCA) not completed/contain significant omissions; Diagnoses not made (due to gaps in diagnostic pathways, e.g., adult autism).	
Inadequate or inappropriate provision: Assessments contain inaccuracies or errors, are out of date, are of poor quality or are non‐compliant with local and/or national guidelines, policy or legislation; Diagnostic errors and misdiagnoses.	
Dehumanising and discriminatory provision: Assessments based on stereotypical/prejudicial assumptions (e.g., in relation to people who are racially and ethnically minoritised); Diagnoses made punitively (e.g., personality disorder).	
*‘Delayed assessments—primarily delayed MHA assessments—constituted a sizeable proportion of “Moderate harm” PSIs (33.2%). Outcomes included self‐harm; deterioration; and risks to others:* “*Patient has relapsed into paranoid psychosis and is at high risk of harm to others, property, public, and above all himself —he has lost a great deal of weight, is looking gaunt, failing to eat and refusing all medication*.”’ [45]	
**2. Care and/or treatment (including its planning and co‐ordination):**	
Gaps, absences or omissions in provision: Care/treatment not available or provided (e.g., inpatient care/treatment; care co‐ordination/CPA/CMHT, care/treatment that is culturally appropriate, routine monitoring of medication side effects).	[13, 39, 42, 44, 45, 46, 52, 53, 54, 56, 57]
Inadequate or inappropriate provision: Duration and/or intensity not appropriate to meet needs; Non‐compliance with the CPA, MHA or NICE guidelines; Misappropriation of the MCA/capacity to justify no or poor care/treatment; One‐size‐fits‐all approach (e.g., lack of personalisation and choice); Lack of continuity (e.g., multiple care co‐ordinators); Lack of involvement of person/caregiver/family.	
Dehumanising and discriminatory provision: Care/treatment that is punitive or shaped by stereotypical/prejudicial assumptions; Care/treatment that involves an abuse of power (e.g., making care/treatment conditional on professionally defined behaviours).	
‘*When I was working in the crisis team there was just so much going on and there would only be two of us on the shift. We would go to people's houses and literally do a, “Are you alive? Yes, you are. Are you taking your medication? Great, here's some more, okay, we have to go.” (Registered Nurse 2)'*[39]	
**3. Access/Admission (to provision):**	[3, 13, 30, 39, 40, 42, 43, 44, 45, 46, 48, 52, 53, 54, 55, 56]
Gaps, absences or omissions in provision: No provision available to access (e.g., evenings and weekends or for people who are racially, ethnically and culturally minoritised); Failure to accommodate individual access needs (e.g., no home visits, self‐referral only, accepting non‐engagement); Gatekeeping and other strategies to manage demand (e.g., high or low acuity/risk thresholds).	
Inadequate or inappropriate provision: Access is delayed (e.g., due to waiting lists, delayed referrals, missed diagnoses, restricted out‐of‐hours provision).	
Dehumanising and discriminatory provision: Exclusion from provision/access due to stereotypical/prejudicial assumptions (e.g., exclusion on the basis of diagnosis or behaviour, perceptions about who is deserving versus undeserving of care, racism); Failure to accommodate access needs arising from aprotected characteristic; Access is withheld/conditional (e.g., abuse of power).	
*‘“Terribly [impact of waiting times]. My daughter did not receive psychological support according to national guidelines. After waiting four months for first appointment, she only received two further appointment before her death. Too little, too late” (R44, female, carer, aged 40–59)*. [52]	
**4. Discharge and transitions of care:**	[40, 42, 44, 45, 46, 47, 52, 57]
Gaps, absences or omissions in provision: Gaps in hospital discharge planning (e.g., no assessment of risk, or of needs in the community, no handover to community teams); No follow up following discharge from hospital.	
Inadequate or inappropriate provision: Discharge despite unmet needs (e.g., of people labelled ‘hard to engage’); Uncoordinated transitions of care (e.g., from an out of area placement); Non‐compliance with local and/or national policies; Errors/inaccuracies in discharge planning (e.g., risk assessment is out of date); Mode of discharge planning not appropriate (e.g., not face to face).	
Dehumanising and discriminatory provision: Punitive discharge (e.g., of people who self‐harm).	
*‘The ward round held before A's discharge was not attended by any professionals from the CMHT as A's CCO was on leave. The review noted that A was offered a follow‐up appointment, which he did not attend. A missed a series of subsequent appointments. Despite A's CCO recording that he would endeavour to visit A at home, no visits took place. This illustrates failure to adhere to the law, given the statutory responsibility of the CMHT with regard to A's CTO, in addition to being an inadequate response to risk management*.’ [46]	
**5. Communication and documentation:**	[3, 30, 40, 44, 45, 46, 48, 49, 52, 54, 56, 57]
Gaps, absences and omissions in provision: Failure to communicate, document, share information or listen (to/with patients, carers and internal/external staff/organisations); No translation services; Patient records not available or content contain significant omissions.	
Inadequate or inappropriate provision: Content contains errors (e.g., in patient records) or is out of date (e.g., about risks); Delays in information being shared; Ineffective or inadequate handover processes and procedures.	
Dehumanising and discriminatory provision: Communication/documentation is shaped by stereotypical/prejudicial assumptions or is punitive (e.g., in relation to people who self‐harm or have a personality disorder diagnosis).	
‘*It was repeatedly noted in the PFDs that the family members of individuals under the care of mental health services were often denied the opportunity to have any input into their loved one's care. The following examples are illustrative of this: Case 55: Due to inadequate communication of potentially significant information between [the deceased's] family and staff members, [the deceased] was put at risk*.’ [48]	

**Abbreviations:** CCO: Care Co‐ordinator; CMHT: Community Mental Health Team; CPA: Care Programme Approach; CTO: Community Treatment Order; MCA: Mental Capacity Act; MHA: Mental Health Act; NICE: National Institute for Health and Care Excellence; PFD: Prevention of Future Deaths; PSI: Patient Safety Incident.

#### 3.3.3 Who Does Institutional Abuse and Neglect Affect?

Specific populations with mental health needs recurrently appeared to be subject to the phenomena, including people who: self‐harm [3, 13, 43, 46, 47]; have been given a personality disorder diagnosis [13, 30, 39, 40, 42, 43, 44, 46, 48, 52, 53, 54]; experience alcohol or substance misuse [3, 30, 40, 44, 46, 48, 52, 54]; are deemed too complex for primary care yet not complex enough for secondary care [13, 39, 44, 46, 52]; require crisis care, MHA assessments and inpatient treatment [40, 42, 43, 44, 46, 48, 53]; and people who do not self‐advocate or have others to advocate on their behalf [40, 42, 44, 46, 47, 52]. Less frequently, papers reported on the phenomena in relation to people who are racially and culturally minoritised [44, 46, 56] and people who avoid or reject services, labelled ‘difficult to engage’ or are perceived to be someone who ‘self‐neglects’ [3, 46, 47]. Isolated examples of the phenomena in relation to people who are perceived to use A&E frequently [54], people with co‐occurring physical health conditions [48], as well as people who are in contact with the criminal justice system [46] were identified.

#### 3.3.4 What Harms Are Associated With Institutional Abuse and Neglect?

There were differences in the severity of harms identified (Table 5), with the severest outcomes consisting of the death of a person with mental health needs [13, 39, 44, 46, 48, 49, 50, 51, 52]. Papers by Foss [46] and Deshpande and Sinclair [57] reported on the deaths of others who were victims of a homicide perpetrated by someone with mental health needs. Non‐fatal harms for people with mental health needs consisted of increased illness acuity or distress, self‐harm and suicide attempts [3, 13, 39, 40, 42, 43, 44, 46, 47, 52, 53, 54, 55, 56], detention under the MHA and hospitalisation [13, 42, 46, 47, 52], psychological harms [3, 13, 30, 43, 44, 50, 52, 54, 55, 56] and reduced quality of life [3, 44, 46, 47, 52, 55]. Isolated examples of psychological harms for unpaid caregivers were described [47, 54, 55, 56], with moral injury [44, 53] and burn‐out [39, 40, 53, 54] described in relation to staff.

**Table 5:** Associated harms and examples.

Type of harm	Description/Examples	Contributing papers
**Person with a mental health need:**		
Death	Death due to suicide, ‘self‐neglect’ or the victim of a homicide.	[13, 39, 44, 46, 48, 49, 50, 51, 52, 57].
Exacerbation of mental illness/distress	Increased illness acuity or distress, self‐harm, suicide attempts, detention under the MHA and hospitalisation.	[3, 13, 39, 40, 42, 43, 44, 46, 47, 52, 53, 54, 55, 56].
Psychological harms	Feelings of being punished, violated, abandoned and excluded. Feeling unworthy/undeserving of care. Feelings of failure, shame and guilt. Feeling stigmatised. Feelings of hopelessness and powerlessness. Anxiety and fear about how or if needs will be met in the future. Re‐traumatisation (as services replicate prior abusive experiences). Fears about being reported to Prevent (a UK government programme that involves reporting people who are susceptible to radicalisation) or having children removed.	[3, 13, 30, 43, 44, 50, 52, 54, 55, 56]
Decreased quality of life	Loss of, or reduced, ability to work, study or maintain basic needs (e.g., eating and washing). Homelessness, loss of housing and unstable housing. Lost life opportunities and altered life trajectories.	[3, 44, 46, 47, 52, 55].
Other	Contact with the police (e.g., in a mental health crisis).	[13, 43, 46, 47]
	Victim of a crime (e.g., abuse, discrimination and exploitation).	[30, 47]
	Medication‐related physical harm (e.g., through lack of monitoring).	[44]
	Development of harmful coping strategies (e.g., substance use).	[55]
**Other people (e.g., staff and informal caregivers/family)**:		
Death	Victim of a homicide.	[46, 57]
Physical harm	Victim of violence or aggression.	[13, 44, 46]
Psychological harms	Staff—moral injury, burn‐out and sickness.	[39, 40, 44, 53, 54, 56]
	Informal caregiver—feeling anxious, distressed, stigmatised; feelings of guilt and shame; decreased well‐being.	[47, 54, 55, 56]

#### 3.3.5 Why Does Institutional Abuse and Neglect Happen?

Potential causal factors were identified at national (macro), institutional (meso) and individual (micro) levels (Tables 6, 7, and 8). Resources, which we interpreted broadly to include financial, material and human resources, was the most frequently identified factor. On a national level, this included the chronic national underfunding of mental health services, coupled with the wider impacts of a programme of austerity on public finances [30, 40, 45, 48, 52]. Broad references to a lack of resources at an institutional level were made repeatedly, as well as to the immediate and proximal consequences of national underfunding (e.g., a shortage of beds and unmanageable caseloads) [39, 40, 42, 44, 45, 50, 52, 53, 54, 55, 56, 57].

Policy factors were noted at both a national [42, 44, 47, 48, 49] and an institutional level [39, 42, 44, 46, 47, 51], including gaps and inadequacies in national legislation, policy and/or guidance (e.g., the MCA and MHA). At an institutional level, workforce issues such as limited supervision, inadequate training and inexperienced leadership were highlighted [13, 30, 39, 40, 42, 44, 45, 46, 48, 56, 57]. Cultural factors at an institutional level included closed safety/safeguarding practices [30, 40, 41, 42, 43, 44, 49, 50], structural and relational fragmentation [3, 30, 39, 42, 44, 45, 47, 48, 49, 50, 53, 54], as well as dehumanising social norms [3, 41, 43, 44, 52, 56].

At an individual level, workforce factors included inadequate knowledge, skills and/or experience, as well as sickness, burn‐out and stress, including feelings of powerlessness, apathy, disillusionment and poor locus of control [13, 30, 39, 44, 45, 53, 54, 56, 57]. These individual workforce factors were linked to hardened and poor attitudes towards patients and harmful relational practices [30, 41, 44, 52, 54]. Individual factors relating to the person with a mental health need were also reported, including high acuity and complexity (exacerbated by gaps and inadequacies in provision), as well as service avoidance and rejection due to previous harmful, traumatic service experiences and apparent ‘non‐adherence’ [13, 30, 42, 43, 46, 47, 56, 57].

**Table 6:** National (macro) potential causal factors.

National (macro) potential causal factors, examples and illustrative quotes	Contributing papers
1. **Resources**: Long‐term underfunding of public mental health services; Impact of austerity on public services and infrastructure (e.g., housing, policing and national infrastructure).	[30, 40, 42, 45, 48, 50, 52]
*‘Case 105: Chronic underfunding of mental health services is creating a risk to life.’* [48] *‘Risk of vulnerability [to disability hate crime] was felt to be compounded by the broader context of the socioeconomic effects of austerity. Participants referred to reductions in support packages, absence of preventative support and difficulties with accessing services….’* [30]	
2. **Policy**: Inadequacies and gaps in national legislation, policy and statutory guidance (e.g., the MCA, MHA and the CQC's regulatory requirements); Failure of national organisations (e.g., NHS England, Department of Health and Social Care) to learn from serious incidents (e.g., generic, defensive and deflective responses to PFDs reports); National policy necessitating major reorganisation to implement organisationally (e.g., payment by results and clustering).	[39, 42, 44, 47, 48, 49]
*‘The service went through various transformations which meant that once the service boundaries became tighter and more clear, by the time people got to us they were very, very ill. So had we have caught them a bit sooner, they might not have needed necessarily our level of input but because of clustering and Payment by Results, we just ended up with very unwell people… (Registered Nurse 2).’* [39] *‘The presumption of capacity [in the context of the Mental Capacity Act] should be rebuttable, but in the absence of any clear and effective mechanism for challenge by significant individuals in the adult's life, it becomes irrebuttable and dangerous.’* [47].	

**Abbreviations:** CQC: Care Quality Commission; MCA: Mental Capacity Act; MHA: Mental Health Act; PFDs: Prevention of Future Deaths.

**Table 7:** Institutional (meso) potential causal factors.

Institutional (meso) potential causal factors, examples and illustrative quotes	Contributing papers
1. **Resources:** Lack of financial resources with consequential gaps and inadequacies in commissioned material and human resources (e.g., insufficient inpatient beds, inefficient/incompatible IT systems, under and unsafe staffing [number, skill mix and experience] and resultant recruitment and retention difficulties).	[39, 40, 42, 44, 45, 46, 48, 51, 53, 55, 56, 57].
‘*Lack of resources result in service users being discharged from inpatient settings to community services that are unable to manage risk safely and provide continuity of care. Professional #116.’* [40] ‘*Additional system‐drivers of harm included chronic workforce underinvestment. Staffing shortages seemingly arose from long‐term disinvestment from community teams, causing unmanageable workloads, which damaged recruitment and retention efforts. Whilst inpatient services are limited by bed availability, community‐based providers experienced uncapped caseloads, exceeding safe limits….’* [44]	
2. **Workforce leadership and management**: Poor/inexperienced line management and leadership at the service/team level; Diffuse lines of responsibility and accountability; Lack of opportunities for high‐quality supervision and training.	[13, 30, 39, 40, 42, 44, 45, 46, 48, 56, 57]
‘*…the review discussed that the AMHP did not have access to suitably qualified supervisory support, with knowledge of the law or the AMHP role. Had this support been available, it may have assisted the AMHP in balancing options and risks, considering legislative requirements of the MHA and Code of Practice and intersections with Bert's human rights, particularly Art.2 (Right to life, due to Bert's level of risk)….’* [46]	
3. **Policy**: Local policies or procedures confining staff‐patient time, who can access a service, or limiting the exchange of information between teams and individuals; Local policies that cannot be implemented (e.g., lone working); Referral procedures not fit for purpose; Failure to implement evidence‐based practice guidelines.	[39, 42, 44, 45, 46, 47, 51]
‘*Case 67: There is no ability to admit [a patient with addiction issues] into a “safe space” when in crisis for care and supervision. Essentially patients with dependence are thus discriminated against by psychiatric services, with addiction being regarded as a personal choice.’* [48]	
4. **Service organisation**: Complex, fragmented pathways and systems of care (e.g., multiple services and/or providers, multiple transitions between services/providers with incompatible systems) resulting in communication, collaboration and continuity of care challenges.	[13, 30, 39, 45, 46, 47, 48, 54, 57]
‘*…the fragmented nature of care provision necessitated communication between multiple providers: My team have to spend a lot of time communicating with external agencies and organisations to make sure that the care that's going in is what they've contracted them to do and what they're doing is safe and appropriate and that any concerns are fed back. So it's quite a complex way of doing it. (Clinical Lead Community, Occupational Therapist).’* [39]	
5. **Culture—Closed safety/safeguarding culture**: Organisations that do not learn from incidents and harms; Inaccessible complaints and concerns processes; Widespread fear of blame or punishment; Apathy about the ability of investigations or complaints to enact positive change; Perceptions of ‘safety’ limited to physical safety.	[3, 30, 40, 41, 42, 43, 44, 49, 50]
*‘If you raise any issues or challenge any decision you are seen as a difficult patient. You are not expected to have a valid viewpoint. They know best. They can make life very difficult, refuse to help you, and most likely change your diagnosis to personality disorder so that no one will want to treat you.’ (Service user #5)* [41] *‘Blame cultures’ in mental health and social work could mean that practitioners are afraid to take responsibility or whistle blow for fear of reprisal.* [30]	
6. **Culture—Fragmented culture:** Normalised poor collaboration and co‐operation between services, providers, professionals, service users and caregivers; Team working and partnership working not valued or prioritised.	[3, 30, 39, 42, 44, 45, 47, 48, 49, 50, 53, 54]
*‘Impacts of service divisions were evident. It seemed that providers avoided delivering interventions requiring between‐service multidisciplinary collaboration, including physical healthcare provision for people with severe mental illness. There was a tendency for siloed working, meaning that service users' care was not cohesive: 90% of the people in my clinic will be on dreadful polypharmacy … the burden of side effects is horrendous, the interactions are dreadful, and the problem is, nobody will take responsibility and technically it's not my job either. (Pharmacist, Specialist medication clinic, individual interview).’* [44]	
7. **Culture—Dehumanising culture:** Normalised relational practices that exacerbate power imbalances and retraumatise; Normalised stigmatising and discriminatory attitudes, including structural racism.	[3, 41, 43, 44, 52, 56]
*‘Ultimately, every interaction pummelled into me the understanding that I had no control, no agency, no right to have my needs met. I was not important. I did not matter. I was a thing, and throughout all my interactions was reminded that I needed services, but they could choose to drop me at any moment. My life became about them, about avoiding their punishment and anger, about playing their games to find the mythical reward of recovery.’* [3]	

**Abbreviations:** AMHP, Approved Mental Health Professional; IT, Information Technology.

**Table 8:** Individual (micro) potential causal factors.

Individual (micro) potential causal factors, examples and illustrative quotes	Contributory papers
1. **Workforce**: Burn‐out, stress and sickness; Hardened and poor attitudes towards patients leading to harmful relational practices; Lack of cultural awareness/competence; Inadequate knowledge, skills and experience.	[3, 13, 30, 39, 40, 42, 43, 44, 45, 47, 53, 54, 56, 57]
‘*Practitioners wanted to help but felt powerless. They recognised complex, long‐standing problems but did not believe they could meet someone's needs or help them to stop self‐harming. Negative attitudes when people re‐attend were linked to powerlessness and frustration. Experience of burnout was described… as becoming “hardened” or “cold” towards patients—which may in turn exacerbate feelings of worthlessness by patients*. [54]	
2. **Person with mental health needs**: High acuity and complexity due to gaps and inadequacies in service provision; Service avoidance and rejection due to previous harmful, traumatic service experiences; Specific diagnoses or behaviours that elicit a neglectful response from service providers (e.g., personality disorder); So called ‘non‐adherence’ with treatment and services.	[3, 13, 30, 42, 43, 46, 47, 56, 57]
‘*Unhelpful or distressing encounters with community‐based mental health services may cause service users to feel unsafe when using these services. Similarly, prior experiences of compulsory treatment under Mental Health Act legislation may erode trust in care systems, potentially leading service users to conceal important risk information from care teams.’* [13]	

## 4 Discussion

### 4.1 Summary

This scoping review aimed to identify and describe the peer‐reviewed evidence on the phenomena of institutional abuse and neglect in UK community mental health services, for adults of working age. Our search did not identify evidence explicitly aiming to investigate our phenomena of interest, but 22 papers reporting on 21 discrete studies (or works) met our inclusion criteria. The included evidence was primarily published from 2018 onwards and was exploratory and descriptive in nature. Whilst the phenomena are grounded in safeguarding and social care policy, practice and legislation, only a minority of papers derived from this discipline (*n* = 3) [30, 46, 47], with the majority deriving from the fields of patient safety (*n* = 8) and health services and delivery research (*n* = 7). We did not identify any prior definitions or conceptualisations of the phenomena (our second review question). Pragmatically, we developed our third review question and sub‐questions post hoc to advance conceptual knowledge about the phenomena's characteristics (*When does it happen? How does it happen? Who does it affect? What harms are associated with it?)* and potential causal factors (*Why does it happen?)*.

### 4.2 Contribution to Knowledge and Discussion of Findings in the Context of Existing Knowledge

Our descriptive synthesis enabled us to identify institutional *neglect* as the primary phenomenon described, with access or admission to treatment or assessment the institutional provision/process most frequently associated with a failure to meet needs. This aligns with the broader evidence on access and waiting times in UK mental health services, where increased demand and reduced financial investment have caused long delays. For instance, as of 2024, over 1 million people were waiting for mental health services in England, with 345,000 waiting over a year [58, 59]. Access to inpatient mental health treatment has also become more difficult due to fewer beds compared to other Organisation for Economic Co‐operation and Development (OECD) nations, raising admission thresholds and leading to inappropriate discharges [60]—another provision/process associated with institutional abuse and neglect identified by this review.

Multiple harms were identified, including death by suicide, homicide or ‘self‐neglect’. These severest harms were not always described by the patient safety evidence, specifically papers with a dual focus on inpatient and community settings [40, 41, 42, 43]. Harms or ‘safety’ concerns in the community were often compared by these studies' participants to harms in hospital settings, which they appeared to perceive as more severe. This is at odds with evidence attesting to increased suicide rates amongst people under crisis resolution home treatment teams compared to those in inpatient settings [61]. Our findings, therefore, highlight the need for research with a specific focus on community settings and their unique safeguarding and safety challenges and harms [13]. Significantly, the Care Act (2014) and its statutory guidance do not describe the nature or severity of harms it considers within its remit. Our review, consequently, advances knowledge about the breadth of harms (in nature and degree) associated with the phenomena, including increased illness acuity or distress, detention under the MHA, reduction in quality of life, criminalisation and psychological harms. Further, iatrogenic psychological or emotional harms associated with community mental health services have historically been neglected by the peer‐reviewed literature [3]. However, there is an emerging peer‐reviewed evidence base led by researchers with lived experience of mental illness or distress attesting to this phenomenon [3, 30, 32], which this paper joins.

New insights have been generated about the population affected by the phenomena, including community living adults who have been given a personality disorder diagnosis, who self‐harm, and/or who have difficulties with alcohol or substance misuse. These findings align with the broader evidence on poor care experiences and outcomes for such populations [62, 63, 64, 65]. Additionally, people labelled as ‘non‐engaging’, ‘service avoidant’ or ‘self‐neglecting’ were impacted [6, 46, 47]. Historically, some individuals labelled as such may have had their healthcare needs met by Assertive Outreach Teams (AOT) [66]; however, many services in England de‐invested in AOT, reflecting NHS England policy shifts to time‐limited episodes of care, and a Randomised Controlled Trial (RCT) that concluded that AOT does not reduce hospital admissions [67, 68]. Recent preventable homicides [10, 11] have prompted NHS England to advocate for renewed investment in assertive care models [69, 70], a call supported by this review.

The chronic underfunding of mental health services at the macro, national level was the principal causal factor identified, manifesting at an institutional, meso level (e.g., a shortage of beds and inadequately staffed community services) and individual, micro level (e.g., staff burn‐out and hardened attitudes towards patients). The inadequate statutory funding of mental health services has been consistently highlighted by others [5, 71, 72], and this study adds to this body of evidence by identifying its association with institutional abuse, neglect and harm. Whilst resources are discussed in the context of institutional abuse and neglect in existing safeguarding guidance, this is at the institutional level (e.g., the mismanagement of resources by staff leading an organisation) rather than in relation to national underfunding [27, 73]. Further, we identified potential causal factors that related to the fragmented and complex organisation of mental health services, which are typically provided by multiple institutions. This is at odds with current statutory guidance that implies that solo institutions are the perpetrators of institutional abuse and neglect [27].

### 4.3 Evidence Gaps

Our fourth review question aimed to identify gaps in the peer‐reviewed evidence. Besides Kiely and Warnock [74] (discussed below), we found no papers explicitly focused on our phenomena and context. This reveals a significant gap in evidence to inform safeguarding practices in the UK. Given the lack of specific statutory guidance and historical connections with inpatient and residential settings, there is a need to build upon and validate this review's preliminary conceptualisation [27]. Future research should determine the extent of the phenomena and explore potential solutions or preventative strategies.

Gaps in relation to the broader body of knowledge about harms and safety in UK community mental health services were noted. Geographically, there is a bias towards England, with gaps existing in relation to other UK nations which have differing safeguarding legislation and health and social care systems. Methodologically, there was an absence of any intervention or experimental research, with papers primarily exploratory and descriptive in nature, indicating that this body of knowledge is in its infancy.

Multiple potential gaps in relation to the population affected were noted. For example, evidence has been amassing about the inequities that people who are racially minoritised face in relation to mental health outcomes, experiences of, and access to, mental health services [75, 76, 77]. However, only three papers described race or ethnicity in relation to the phenomena of interest [44, 46, 56]. Further, inequalities have been described in relation to sexual and gender minorities; however, we did not identify this in our evidence [77, 78]. Aside from a personality disorder diagnosis and substance misuse, there appears to be a gap in relation to other specific diagnoses. For example, people living with longstanding eating disorders who have not received appropriate interventions who are being placed on palliative care pathways [79]. Or people who have a Serious Mental Illness (SMI) diagnosis, traditionally the primary recipients of long‐term support from CMHTs and the Care Programme Approach (CPA) [80], who are being routinely discharged or ‘off rolled’ to the care of their GP [81, 82].

Whilst our focus when conducting this review was on people with mental health needs themselves, we did extract data on harms, where mentioned, in relation to others. There was a notable gap about harms to informal caregivers who are often left to support and advocate for family members who are acutely unwell alone [44, 54, 55, 83], and harms to families and communities bereaved following a preventable homicide [84]. Whilst noted briefly by Averill and Vincent [13] and Averill and Bowness [44], evidence on harms associated with violence and aggression that do not result in a homicide was largely absent, as was evidence on medication‐related harms resulting from institutional failures, for example, a lack of side‐effect monitoring provision [85, 86]. Further, harms relating to interventions driven explicitly by organisational financial constraints, for example, systematic intervention programmes or care pathways that involve witholding care or prosecuting high‐intensity users of statutory services or people who are recurrently suicidal in a public place [16, 87], were identified as gaps.

### 4.4 Revisiting Terminology

We started this review by acknowledging that there is a lack of consensus surrounding terminology in this emerging field (i.e., our use of the word institutional rather than organisational). Our findings in relation to potential causal factors at the national and broader institutional (meso) level perhaps raise more uncertainties about terminology, since they challenge the notion portrayed in UK statutory guidance that solo institutions are the perpetrators of institutional abuse and neglect [27]. This portrayal is at odds with the reality and complexity of modern‐day mental health services, which are delivered overwhelmingly in the community through a complex web of institutions, providers and organisations. It is also at odds with the influence national policy factors (e.g., funding) have, for example, in determining the nature and extent of what institutions can provide in the context of increasingly confined resources. This review indicates that perhaps alternative language needs to be considered when appropriate, to refer to instances of abuse, neglect or harm that result from factors at the national (macro) level or broader institutional (meso) level. For example, systemic may more accurately reflect harms arising from the complex structure and organisation of community mental health services at the meso level, whilst structural may be more apt to describe harms arising from national factors, including underfunding.

### 4.5 Limitations

It is important to highlight the limitations of this study when considering our findings. Grey literature, for example, SARs or Inquest reports, which are likely to contain pertinent detailed information of relevance to the review topic, were not eligible for inclusion. We confined our search to papers relating to the UK, and an international search may have identified additional papers of relevance. Evidence included in this review was primarily of a descriptive and observational nature, and therefore our review is based on associations and explanations made by participants (where applicable) and/or authors, rather than from highly powered quantitative studies that sought to generate data about causality.

We tied our definition of institutional abuse and neglect to evidence of harm (e.g., self‐harm, suicide and psychological harm). A theoretical paper of significance [74] was excluded on this basis; however, it was the only paper screened during this review that defined and/or aimed to investigate (or theorise about) institutional *neglect* and is therefore worth describing briefly. This study was not underpinned by the Care Act (2014), rather sociological theories about state violence under austerity, yet its definition of institutional *neglect* (*a* ‘*fail[ure] to provide for care needs which they [the institution] have historically recognised as valid*’ [p317]) aligns broadly with our definition (Supporting Material 1). However, it appears to confine what constitutes institutional *neglect* by making an institution's historical provision the benchmark upon which institutional neglect in the present is determined. The paper offers important insights into when, how and why institutional neglect occurred in relation to accessing a LA mental health service, which again broadly aligns with our findings. However, beyond access, further institutional provisions/processes (e.g., discharge and care/treatment) were not the focus of this paper. Austerity, however, was discussed as the primary contributory factor, which aligns with our findings on resources as potential causal factors.

We included papers that clearly articulated potential institutional causal factors, in line with our phenomena of interest. Papers with a focus on adverse events or harms associated with specific interventions (e.g., medication or psychological therapies), national policy and legislation (e.g., CTOs), or the practice of individual clinicians, without evidence of potential institutional causal factors were not included.

## 5 Conclusion

To our knowledge, this is the first review that has synthesised the peer‐reviewed evidence on the topic of institutional abuse, neglect and harm in UK community mental health services for working‐age adults. It has exposed a significant gap in this evidence, including from a social care and safeguarding perspective, where the concept has real‐world practice and legal implications for people with mental health needs, safeguarding practitioners and commissioners. By synthesising evidence from the broader body of knowledge about harms and patient safety in UK community mental health services, we have advanced conceptual knowledge about the phenomena. This study provides a robust foundation for future research endeavours, which should seek to build on our preliminary conceptualisation, identify the phenomena's prevalence, and develop and evaluate preventative strategies to inform real‐world safeguarding and patient safety policy and practice.

### 5.1 Use of Artificial Intelligence Generated Content

AI was not used to generate any original content in this manuscript. Microsoft Copilot was utilised to assist with manuscript brevity and ease of reading.

## Author Contributions

**Bethan M. Edwards:** conceptualisation, formal analysis, funding acquisition, investigation, methodology, project administration, supervision, validation, writing – original draft preparation, writing – review and editing. **Alan Meudell:** formal analysis, investigation, methodology, validation, writing – review and editing. **Ellen Thomas:** conceptualisation, validation, writing – review and editing. **Eva Broeckelmann:** formal analysis, validation, writing – review and editing. **Eva Roberts:** formal analysis, validation, writing – review and editing. **Mark Farmer:** formal analysis, validation, writing – review and editing. **Naomi Ghafoor:** formal analysis, validation, writing – review and editing. **Sarah Markham:** formal analysis, validation, writing – review and editing. **Catherine A. Robinson:** conceptualisation, funding acquisition, methodology, supervision, validation, writing – review and editing. **Angela Sweeney:** methodology, supervision, validation, writing – review and editing. **Sarah Carr:** conceptualisation, funding acquisition, methodology, supervision, validation, writing – review and editing. **Michael Clark:** conceptualisation, funding acquisition, methodology, supervision, validation, writing – review and editing.

## Ethics Statement

Ethical approval was not required for this systematic scoping review.

## Consent

This manuscript did not involve recruiting participants or patients. It is a systematic scoping review, and therefore, consent from participants was not required.

## Conflicts of Interest

The authors declare no conflicts of interest.

## Supporting information


**Supplementary material 1:** Inclusion and exclusion criteria. **Supplementary Material 2:** Ovid Medline search strategy.

## Data Availability

Data sharing is not applicable to this article as no datasets were generated or analysed during the current study.
