# Supplementary material for: Institutional Abuse, Neglect and Harm in UK Community Mental Health Services: A Scoping Review of the Peer‐Reviewed Evidence
Source: Health Expect. 2025 Sep 27;28(5):e70403. doi: 10.1111/hex.70403 (PMC12476028; doi:10.1111/hex.70403)
Supplement: Supplementary file 1 — Supplementary material 1: Inclusion and exclusion criteria. Supplementary Material 2: Ovid Medline search strategy. [file HEX-28-e70403-s001.docx]

***Supplementary material 1: Inclusion and exclusion criteria***

|  | **Include** | **Exclude** |
| --- | --- | --- |
| **Population (Participants)** | Evidence reporting on adults of ‘working’ age (18-66, inclusive) who have mental health needs, living in the community. ‘Mental health needs’ will be defined as: -   - A mental health diagnosis provided by a health or social care professional. - Symptoms indicative of a mental health diagnosis / need e.g., suicide and self-harm. - Accessing or attempting to access services (inclusive of health, social care, emergency services) for mental health needs.   Evidence that reports on the phenomena of interest (below) in this population and in the specified context (below) but includes participants who may be health or social care professionals (for example), or does not include participants (e.g., evidence syntheses, theoretical papers), will also be eligible for inclusion. | Evidence reporting on:-   - Older adults, age 67 or over; children, age 17 or under; or reports a mean age of less than 17 or more than 67. - Adults living in a residential care (home), in a prison setting, or who are an inpatient in hospital. - Adults with a diagnosis of dementia, other neurodegenerative condition, a learning disability, or a neurodevelopmental condition (e.g. Autism or ADHD) if they do not have a co-morbid mental health need as defined. |
| **Phenomenon (Concept)** | Evidence that reports on the phenomena of institutional (organisational) abuse and / or neglect associated with harm.   - *Institutional* was defined using the micro-meso-macro framework at the meso level (Fulop & Glenn, 2015; Richter & Dragano, 2018). Mental health and social care commissioners and providers, including Integrated Care Boards (ICBs), Clinical Commissioning Groups (CCGs), Local Authorities (LA), NHS Trusts and Health Boards are in scope, including the services which they commission or provide. - *Institutional abuse* was defined as acts of abuse that may be sexual, financial, emotional, psychological, physical and discriminatory, as per the Care Act (2014) with evidence of potential institutional causal factors. - *Institutional neglect* was defined as a failure to meet health and care needs in a timely manner with evidence of potential institutional causal factors. - *Institutional causal factors* were defined as causal factors at the institutional / meso level (e.g., leadership, an organisations’ resources, staffing, culture, policies, processes, structure). - *Harm* was defined broadly, inclusive of physical and psychological harms of differing severity e.g., suicide, homicide, deterioration in physical and mental health and readmission. | Evidence reporting on:-   - Intuitional abuse and / or neglect that is not associated with harm. - Abuse, neglect or harm in the absence of institutional causal factors e.g.,:-   - The perpetrator is an individual.   - It arises from a particular intervention (e.g., psychological therapy, psychiatric medication), without evidence of institutional causal factors.   - It arises from national policy/legislation (e.g., Care and Treatment Orders [CTOs]), without evidence of institutional causal factors. |
| **Context** | Evidence that reports on the context of ‘open’ community or outpatient based mental health and social care services in the UK. This includes:-   - NHS services or NHS commissioned services: community mental health teams, crisis teams, mental health liaison services, IAPT, community psychology. - Social care services or social care commissioned services: support at home funded through personal budgets, direct payments, or the local authority, e.g., support work, social work, day services, reablement services, charitable / community groups.   Abuse and / or neglect may occur in (but not limited to) a hospital outpatient setting, home, in a public place, over the internet and over the phone. | Evidence reporting on:-   - Residential care setting or care home or 24 hour supported housing setting. - Prison services, inpatient hospital services, MHA ‘places of safety’. - Non-UK based services. |
| **Date** | Published 2000 – onwards, coinciding with the publication of No Secrets or reports on data generated post 2000. | Published prior to 2000 or reports on data generated before 2000. |
| **Language** | English | Not in English |
| **Country** | Evidence reports on data generated in the UK or if data was not generated, the contents is relevant to the UK. | Evidence reports on data generated outside the UK or if data was not generated, contents are not relevant to the UK. |
| **Information / Evidence type** | Peer reviewed publications, inclusive of primary studies (any methodology), evidence syntheses (any methodology), as well as theoretical and non-empirical publications (e.g., editorials, conceptual articles). | - Blogs, conference abstracts, conference presentations, Theses, books. - Grey literature, namely policy documents, guidelines, and briefings. |

***Supplementary Material 2: Ovid Medline search strategy***

| 1. | "mental health".tw. |
| --- | --- |
| 2. | (psychiatry or "mental illness" or "psychiatric illness" or "mental disorder*" or "psychiatric disorder*" or "mental health condition*" or "psychiatric condition*" or "mental health problem*" or "psychiatric problem*" or "mental ill health" or "mental distress" or "emotional distress" or "serious mental illness" or SMI).tw. |
| 3. | (anxiety or "anxiety disorder*" or agoraphobia or "neurotic disorder*" or neurosis or "obsessive-compulsive disorder" or OCD or phobia or "panic disorder*").tw. |
| 4. | "dissociative disorder*".tw. |
| 5. | "hoarding disorder".tw. |
| 6. | ("affective disorder*" or "mood disorder*" or "bipolar disorder*" or bipolar or "manic depression" or depression or "depressive disorder*").tw. |
| 7. | ("postpartum depression" or "prenatal depression").tw. |
| 8. | ("premenstrual dysphoric disorder" or PMDD).tw. |
| 9. | ("seasonal affective disorder" or SAD).tw. |
| 10. | ("feeding and eating disorder*" or "eating disorder*" or anorexia or "anorexia nervosa" or "binge-eating disorder" or bulimia or "bulimia nervosa" or "eating disorder* not otherwise specified" or EDNOS).tw. |
| 11. | "personality disorder*".tw. |
| 12. | (psychosis or psychoses or psychotic or "psychotic disorder*" or schizophrenia or "schizoaffective disorder" or schizoaffective or "delusional disorder" or paranoia).tw. |
| 13. | (("substance abuse" or "drug abuse" or "alcohol abuse" or alcoholism or addiction) adj1 (drug* or alcohol)).tw. |
| 14. | ("post-traumatic stress disorder" or PTSD or "complex post-traumatic stress disorder" or CPTSD).tw. |
| 15. | ("adult attention deficit hyperactivity disorder" or "adult ADHD" or "adult attention deficit disorder" or "adult ADD").tw. |
| 16. | ("self-harm" or "self-injurious behaviour" or "self-mutilation" or "self-inflicted injury" or overdose).tw. |
| 17. | (suicide or suicidal).tw. |
| 18. | "psychosocial disabilit*".tw. |
| 19. | exp Mental Health/ |
| 20. | exp Mental Disorders/ |
| 21. | exp Self-Injurious Behavior/ |
| 22. | exp suicide/ or exp suicidal ideation/ or exp suicide prevention/ or exp suicide, attempted/ or exp suicide, completed/ |
| 23. | 1 or 2 or 3 or 4 or 5 or 6 or 7 or 8 or 9 or 10 or 11 or 12 or 13 or 14 or 15 or 16 or 17 or 18 or 19 or 20 or 21 or 22 |
| 24. | ("organisational neglect" or "institutional neglect").tw. |
| 25. | ("organisational abuse" or "institutional abuse").tw. |
| 26. | ("organisational harm" or "institutional harm").tw. |
| 27. | (neglect* adj2 (organisation* or institution* or service* or team* or provider* or system* or practice*)).tw. |
| 28. | (abus* adj2 (organisation* or institution* or service* or team* or provider* or system* or practice*)).tw. |
| 29. | (harm* adj2 (organisation* or institution* or service* or team* or provider* or system* or practice*)).tw. |
| 30. | (iatrogenic or iatrogenesis or "iatrogenic harm*").tw. |
| 31. | (wrongdoing* or malpractice* or "poor practice*" or "unethical practice*" or "misconduct" or "negligence" or negligent or "dereliction of dut*" or "abuse of power*" or "abuse of trust" or "abuse of position" or "ill-treat*" or maltreat* or mistreat*).tw. |
| 32. | (abus* adj1 (psychological* or physical* or sexual* or financial* or material* or discrimina*)).tw. |
| 33. | (harm* adj1 (psychological* or physical* or sexual* or financial* or material* or discrimina*)).tw. |
| 34. | "act* of omission".tw. |
| 35. | ("safeguarding adults" or "adult safeguarding" or "safeguarding adult* board*" or "adult protection" or "no Secrets" or "serious case review*").tw. |
| 36. | "patient safety".tw. |
| 37. | exp Patient Safety/ |
| 38. | exp "Attitude of Health Personnel"/ |
| 39. | exp Medical Errors/ |
| 40. | exp Iatrogenic Disease/ |
| 41. | exp Malpractice/ |
| 42. | exp Professional Misconduct/ |
| 43. | exp Emotional Abuse/ |
| 44. | exp Physical Abuse/ |
| 45. | "duty of candour".tw. |
| 46. | 24 or 25 or 26 or 27 or 28 or 29 or 30 or 31 or 32 or 33 or 34 or 35 or 36 or 37 or 38 or 39 or 40 or 41 or 42 or 43 or 44 or 45 |
| 47. | ("community mental health" or "outpatient mental health" or "community psychiatry" or "outpatient psychiatry" or "community mental health team*" or CMHT).tw. |
| 48. | ("primary care mental health" or "mental health primary care" or "secondary care mental health" or "mental health secondary care").tw. |
| 49. | ("improving access to psychological therapies" or IAPT or "talking therap*").tw. |
| 50. | ("peer support" or "befriending").tw. |
| 51. | "assertive outreach".tw. |
| 52. | ("early intervention in psychosis" or EIP).tw. |
| 53. | ("early intervention" adj2 psychosis).tw. |
| 54. | ("crisis home treatment team*" or "crisis resolution home treatment team*").tw. |
| 55. | (crisis adj1 (team* or service* or outreach or intervention* or programme*OR support)).tw. |
| 56. | (urgent adj1 ("mental health" or "mental ill health" or psychiatry)).tw. |
| 57. | (emergency adj1 ("mental health" or "mental ill health" or psychiatry)).tw. |
| 58. | ("mental health liaison" or "liaison psychiatry").tw. |
| 59. | ("community social care" or "community care" or "community social service*" or "community social work*").tw. |
| 60. | ("personal budget*" or "individual budget*" or "direct payment*").tw. |
| 61. | ("day centre*" or "day service*").tw. |
| 62. | "reablement service*".tw. |
| 63. | ("section 117 aftercare" or "section 117 support").tw. |
| 64. | "care act assessment*".tw. |
| 65. | ("community psychiatric nurs*" or CPN or "community mental health nurs*" or "community mental health practitioner*" or "community mental health worker*" or "clinical psychologist*" or "psychological wellbeing practitioner*" or "primary care mental health practitioner*" or "lived experience practitioner*" or "peer support worker*" or "community social worker*" or "community psychiatrist*" or "community support worker*").tw. |
| 66. | ("mental health social work*" or "approved mental health professional*" or AMHP or "community support worker*").tw. |
| 67. | exp Community Mental Health Centers/ |
| 68. | exp Community Mental Health Services/ |
| 69. | exp Community Psychiatry/ |
| 70. | 47 or 48 or 49 or 50 or 51 or 52 or 53 or 54 or 55 or 56 or 57 or 58 or 59 or 60 or 61 or 62 or 63 or 64 or 65 or 66 or 67 or 68 or 69 |
| 71. | 23 and 46 and 70 |
| 72. | limit 71 to (english language and yr="2000 -Current") |
